# Pitfalls in identifying active catalyst species

**DOI:** 10.1038/s41467-020-18192-3

**Published:** 2020-09-11

**Authors:** Jiazheng Ren, Yongsheng Chen

**Affiliations:** Energy and Catalysis Laboratory, Department of Mechanical and Automation Engineering, The Chinese University of Hong Kong, Shatin, New Territories, Hong Kong SAR, China

**Keywords:** Catalytic mechanisms, Heterogeneous catalysis, Chemical engineering, Characterization and analytical techniques

**Arising from** Pereira-Hernández, X. I. et al. *Nature Communications* 10.1038/s41467-019-09308-5 (2019)

Catalytic CO oxidation is an important reaction for both applied and fundamental research. Single-atom catalysts (SACs) have received considerable attention in recent years due to its excellent performance in CO oxidation. Pereira-Hernández et al.^1^ prepared Pt SACs using two methods: (1) conventional wet chemical synthesis (strong electrostatic adsorption–SEA) and (2) high-temperature vapor-phase synthesis (atom trapping–AT). As synthesized, both SACs were inactive for low-temperature (<150 °C) CO oxidation. After a treatment in CO at 275 °C, both catalysts showed enhanced reactivity. In particular, the AT catalyst was significantly more active, with onset of CO oxidation near room temperature. The authors claimed that the high reactivity at low temperatures could be related to the improved reducibility of lattice oxygen on the CeO_2_ support based on near-ambient pressure X-ray photoelectron spectroscopy (NAP-XPS) and CO temperature programmed reduction (CO-TPR). The evidence provided in the paper, when properly analyzed, however, does not support this claim. Specifically, CO does not react with ceria at 50 °C, and Pt metal dispersion is significantly higher in the activated AT catalyst than in the SEA, which may well account for the difference in performance between the two catalysts.

The authors intended to use CO-TPR to probe the difference between the two catalysts in the reaction between CO and oxygen from the catalyst. Experimentally, a catalyst sample would have been “activated via CO reduction at 275 °C, then exposed to an oxidative atmosphere (10% O_2_, 30 min) at 200 °C” before a CO-TPR test. They stated that “because both catalysts were exposed to an oxidative treatment prior to the CO-TPR, the CO_2_ must come from reactive oxygen species accessible during reaction”, and subsequently claimed that “the AT catalyst contains ceria sites that are reducible at low temperatures where the AT catalyst is active for CO oxidation”. This conclusion is actually directly contradicted by the mass spectrometry results during the NAP-XPS experiments (Supplementary Fig. 11, note all cited Figures in this Comment are referred to those in the original paper^1^), which clearly show that at 50 °C, no CO_2_ was detected for either the AT or the SEA catalyst when only pure CO was present. In other words, no ceria reduction by CO at 50 °C takes place for both catalysts. The most significant difference in the procedures between the CO-TPR and the NAP-XPS experiments is the additional oxidative pretreatment in CO-TPR that the authors had intended “to remove any adsorbed species and to replenish the oxygen on the support”. Unfortunately, this oxidative treatment would most likely have re-oxidized the metal Pt to Pt oxide, which would subsequently contribute to the low-temperature CO_2_ signals in CO-TPR. In fact, the NAP-XPS results in Figs. 5 and 6 clearly show reduced contents of Pt(0) and Ce(3+) after the catalysts have been treated in CO + O_2_ and pure CO at 50 °C. For example, the Pt(0) content decreased from 82.7 to 62.0% for the AT catalyst. The oxidative pretreatment in the CO-TPR experiments employed a more oxidizing atmosphere (30% O_2_) and a higher temperature (200 °C) than the conditions used in the NAP-XPS experiments. Thus, more Pt oxide is expected to have existed at the beginning of the CO-TPR tests than the extents suggested by the NAP-XPS experiments. Evidently, the low-temperature CO_2_ signals in the CO-TPR experiment are not from the reduction of the ceria support but more likely from the reduction of Pt oxide introduced by the oxidative treatment in the CO-TPR experiments.

The authors performed NAP-XPS on both catalysts and monitored the change in the composition of Ce(3+) species. The SEA catalyst had a negligible change in the amount of Ce(3+) species when exposed to CO and CO oxidation cycles while the AT catalyst showed a more noticeable change (see Figs. 5 and 6). The authors claimed that these experiments demonstrated the facile reaction of surface oxygen with CO adsorbed on the Pt nanoparticles and it helped explain the low-temperature reactivity of the AT catalyst. The inference is again directly contradicted by the mass spectrometry results during the NAP-XPS experiments (Supplementary Fig. 11) as no CO_2_ was detected when only pure CO was present. The observed change in the amount of Ce(3+) species during the CO and CO+O_2_ cycles is probably a direct result of changing O_2_ partial pressure as CeO_2_ is a known oxygen storage material and ready to exchange oxygen with the O_2_ in the surrounding leading to varying Ce chemical state^2^. It may also be a problem with the XPS data analysis. The authors used CasaXPS software to quantify different Ce species with a Shirley background subtraction. The Ce 3d line fitting was carried out according to a model described in two earlier reports in 2009 and 2015^3, 4^. However, the 2009 report cautioned about Shirley background and further stated that decomposing the complicated spectrum is “partly ambiguous in principle”^3^. The 2015 report used a linear background^5^, echoing the CasaXPS Manual on Ce data analysis that “a linear background provided the most reproducible results”^5^.

The authors collected HAADF-STEM images for the activated AT and SEA catalysts, found very similar particle size for them, and concluded the differences in reactivity between the AT and SEA catalysts cannot be attributed to the differences in the Pt particle size and must be related to the catalyst support. However, as reported in the captions of Figs. 5 and 6, the XPS measurements have that the Pt/Ce ratios in the activated AT and SEA catalysts were ~0.030 and ~0.015, respectively. The significantly higher Pt/Ce ratio in the AT catalyst manifests a better Pt dispersion^6^, which is well known to strongly affect catalysis^7^.

There are other technical issues in the work:

1. Isothermal CO oxidation reaction performance at 50 °C in the NAP-XPS setting over the AT catalyst, as probed by the mass spectrometry (Supplementary Fig. 11), clearly shows that the AT catalyst loses virtually all its activity in about 30 min (estimated from the CO_2_ mass profile and the experimental description). Thus, the result undermines the authors’ many claims including the benefits of high-temperature vapor-phase synthesis and a suitable-for-industrial-use catalyst with high thermal stability and high activity for low-temperature CO oxidation. In addition, little performance data have been provided for the AT catalyst. In fact, only 8 data points are shown for the AT catalyst as in Fig. 1, which was a test in <25 min (estimated from a starting temperature at 25 °C, the last temperature <75 °C, and the ramp rate of 2 °C/min). No repeatability/stability data are provided for this catalyst, which are essential for the evaluation of its catalytic performance.
2. As shown in Fig. 5, the Pt(0) content evolves from 82.7%, down to 68.0%, further down to 58.9%, and then back up to 62.0% corresponding to CO, CO + O_2_, CO, and CO + O_2_ atmospheres, respectively. Known chemistry would not be able to explain why there is less Pt(0) when the atmosphere changes from CO + O_2_ to CO (becoming less oxidizing), and there is more Pt(0) when it switched back to CO + O_2_. Similar result is observed for the SEA catalyst as shown in Fig. 6. Moreover, the authors performed a harsh reduction treatment with CO at 450 °C for 8 h to probe the strength of the interaction between Pt and CeO_2_ (Supplementary Fig. 10c, d). For the AT catalyst, 94.5% Pt was reduced to Pt(0) while 18.9% Ce reduced to Ce(3+); Fig. 5 shows a milder reduction treatment of the same catalyst resulting in a plausible 82.7% of Pt(0) and a troubling 28.1% of Ce(3+). These may again be a problem with the XPS data analysis as discussed earlier. The authors should process their XPS data using a linear background, which hopefully would result in consistent other than self-contradicting results.
3. CO oxidation reaction was monitored by DRIFTS on the activated AT and SEA catalysts at 50 °C (see Fig. 3c, d). More gas phase CO_2_ is recorded for the SEA catalyst than the AT catalyst. This is in direct contradiction to the activity measurements that the AT catalyst is active at this temperature while the SEA is not.
4. Calculation of the turnover frequency (TOF). The authors had it right when stating “The TOF was calculated using the number of CO_2_ molecules formed per second (rate) divided by the number of active sites”. However, it is not self-consistent when the active sites were calculated by using the total number of Pt atoms deposited on the ceria surface. On one hand, if the authors truly believe that some ceria sites are determining the low-temperature activity of the AT catalyst, then it would be more logical to use the number of “active ceria sites” in the calculation. On the other hand, if the authors believe the surface metal Pt sites are active as shown in Fig. 7, it is clear that “the total number of Pt atoms deposited on the ceria surface” would not be the right count because it includes single-atom Pt species and inaccessible metal Pt atoms in the bulk.

## Data Availability

The data cited in the current work are all from Pereira-Hernández, X. I. et al.’s work and available at 10.1038/s41467-019-09308-5.

## References

[1] Pereira-Hernández XI (2019). Tuning Pt-CeO_2_ interactions by high-temperature vapor-phase synthesis for improved reducibility of lattice oxygen. Nat. Commun..

[2] Grinter DC (2016). Spillover reoxidation of ceria nanoparticles. J. Phys. Chem. C..

[3] Skála T, Šutarabc F, Prince KC, Matolín V (2009). Cerium oxide stoichiometry alteration via Sn deposition: Influence of temperature. J. Electron Spectrosc..

[4] Kato S (2015). Quantitative depth profiling of Ce^3+^ in Pt/CeO_2_ by in situ high-energy XPS in a hydrogen atmosphere. Phys. Chem. Chem. Phys..

[5] Fairley, N. *CasaXPS Manual 2.3. 15*. (Casa Software Ltd, Teignmouth, 2009).

[6] Niemantsverdriet, J. W. *Spectroscopy in Catalysis: An Introduction*. (John Wiley & Sons, Hoboken, 2007).

[7] Imelik, B. *Metal-Support and Metal-Additive Effects in Catalysis*. (Elsevier, Amsterdam, 1982).

